# Thermal Conductivity of Epoxy Resin Composites Filled with Combustion Synthesized h-BN Particles

**DOI:** 10.3390/molecules21050670

**Published:** 2016-05-20

**Authors:** Shyan-Lung Chung, Jeng-Shung Lin

**Affiliations:** 1 Advanced Optoelectronic Technology Center, National Cheng Kung University, Tainan 70101, Taiwan; 2 Department of Chemical Engineering, National Cheng Kung University, Tainan 70101, Taiwan; N38981232@mail.ncku.edu.tw

**Keywords:** h-BN/Epoxy resin composites, self-propagating high temperature synthesis (SHS), hexagonal boron nitride (h-BN), thermal conductivity

## Abstract

The thermal conductivity of epoxy resin composites filled with combustion-synthesized hexagonal boron nitride (h-BN) particles was investigated. The mixing of the composite constituents was carried out by either a dry method (involving no use of solvent) for low filler loadings or a solvent method (using acetone as solvent) for higher filler loadings. It was found that surface treatment of the h-BN particles using the silane 3-glycidoxypropyltrimethoxysilane (GPTMS) increases the thermal conductivity of the resultant composites in a lesser amount compared to the values reported by other studies. This was explained by the fact that the combustion synthesized h-BN particles contain less –OH or active sites on the surface, thus adsorbing less amounts of GPTMS. However, the thermal conductivity of the composites filled with the combustion synthesized h-BN was found to be comparable to that with commercially available h-BN reported in other studies. The thermal conductivity of the composites was found to be higher when larger h-BN particles were used. The thermal conductivity was also found to increase with increasing filler content to a maximum and then begin to decrease with further increases in this content. In addition to the effect of higher porosity at higher filler contents, more horizontally oriented h-BN particles formed at higher filler loadings (perhaps due to pressing during formation of the composites) were suggested to be a factor causing this decrease of the thermal conductivity. The measured thermal conductivities were compared to theoretical predictions based on the Nielsen and Lewis theory. The theoretical predictions were found to be lower than the experimental values at low filler contents (< 60 vol %) and became increasing higher than the experimental values at high filler contents (> 60 vol %).

## 1. Introduction

As microelectronic devices have become more integrated and denser, heat dissipation has also become an important problem because heat generation can increase the temperature of devices causing fatal damage and induce thermal fatigue that reduced operational efficiency and service life [1,2,3]. To solve this problem, application of high thermal conductivity materials is necessary in addition to the design of devices for heat dissipation. For this purpose, in addition to high thermal conductivity, many other properties such as moisture resistance, adhesiveness, mechanical strength and low temperature processability are often required for these high thermal conductivity materials. Since no single material can meet all these requirements, development of high thermal conductivity composite materials is necessary.

Epoxy resins have been widely used in coatings, adhesives, highly-integrated memory chips, electronic packaging and light emitting diodes (LEDs) because of their chemical stability, adhesive properties, excellent resistance to several solvents, good mechanical properties and superior electrical resistivity [4,5,6]. However, epoxy resins have a poor thermal conductivity (0.15–0.25 W/mK [7,8]). In order to enhance the thermal conductivity, thermally conductive but electrically insulative materials should be introduced to form composite materials. Silica and alumina particles have long been used as fillers for epoxy resin composites in industry. However, the thermal conductivities of these oxides are one to two orders lower than that of some nitrides, *i.e.*, aluminum nitride (AlN) and boron nitride (BN) [9,10,11]. Since AlN is reactive to moisture and water, but BN is chemically stable and inert to environment, BN has been considered as a potential filler candidate for the fabrication of high thermal conductivity epoxy resin composites for industrial applications.

Many studies have been devoted to studying the processing and characterization of polymer composites filled with various inorganic fillers [1,2,12,13,14,15,16,17,18]. For enhancement of the thermal conductivity of epoxy resin by filling with h-BN particles, it has been reported in many studies [16,17,18,19,20,21] that surface treatment can further increase the thermal conductivity of the composites. Due to poor affinity between the naked (untreated) h-BN particles and epoxy resin, pores (or voids) are easily formed at the interface, resulting in a high thermal conduction barrier. These pores (or voids) can be reduced by improving the interface affinity by some type of surface treatment. The interface thermal conduction resistance can thus be reduced, resulting in a further increase in thermal conductivity of the composites. Xu *et al.* [16] prepared h-BN/epoxy resin composites by using 5–11 μm h-BN particles, equiaxial in shape, and obtained a thermal conductivity of 10.3 W/mK at a filler content of 57 vol % with the h-BN particles surface-treated by the silane Z-6040, as compared to 5.27 W/mK when using the same size of h-BN particles but with no surface treatment. By using 0.6–1.2 μm h-BN particles, Gu *et al.* [17] obtained epoxy resin composites with thermal conductivity of 1.052 W/mK or 0.97 W/mK at a filler content of 60 vol % when the h-BN particles were surface-treated with γ-aminopropyltriethoxysilane (KH550) or were not surface-treated, respectively. Wattanakul *et al.* [18] and Kim *et al.* [19] also prepared epoxy resin composites by using h-BN particles with different surface treatments and concluded that surface treatment led to improved wettability of epoxy resin on the treated surface, improving the interfacial adhesion between BN and epoxy resin, and thus increasing the thermal conductivity (by 22%–80%). Besides, the size and shape of h-BN particles were also found to affect the thermal conductivity of h-BN/epoxy resin composites [20,21].

In all the studies mentioned above, however, commercially available h-BN powders were used and inconsistency was often found between their experimental results. Recently, we have developed a combustion synthesis method for the synthesis of h-BN powder [22], which is characterized by low energy consumption, fast reactions, simple processing and low production cost. As mentioned previously, the interface between h-BN particles and epoxy resin presents a major thermal barrier in the heat conduction of composites. Different synthesis methods may produce h-BN particles with different surface properties, which may affect the effectiveness of any surface treatment and create different interface environments, affecting the thermal conduction resistance and thus resulting in different thermal conductivity. Besides, a h-BN powder with a low production cost can significantly boost the practical applications of h-BN/epoxy resin composite materials. We therefore investigate in this work the thermal conductivity of epoxy resin composites filled with h-BN powders synthesized by our newly developed combustion synthesis method. Effects of the surface treatment of h-BN particles with GPTMS, a type of silane, particle size and filler loading on thermal conductivity were investigated and the measured thermal conductivities were compared with theoretical predictions based on the Nielsen and Lewis equation.

## 2. Results and Discussion

### 2.1. Examination of Surface Treatment

**Figure 1:**
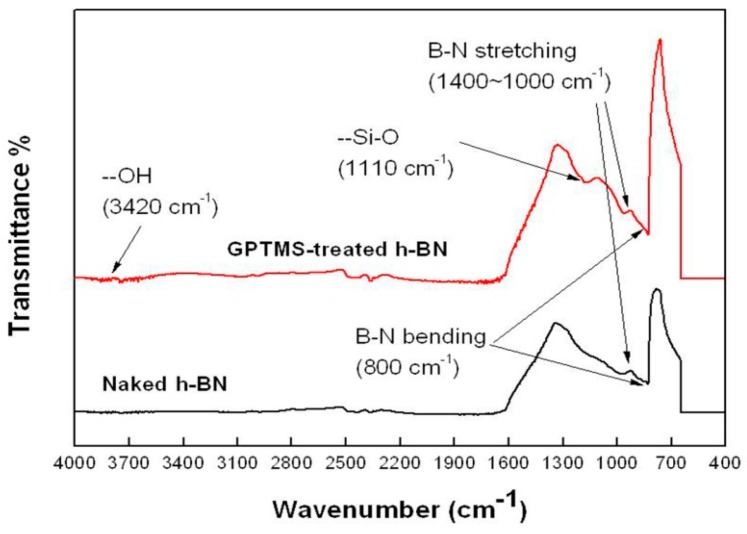
FT-IR spectra of the 2.4 wt % GPTMS-treated and the naked h-BN powders with an average particle size of 10.6 μm.

Figure 1 shows the FT-IR spectra of the 2.4 wt % GPTMS-treated and naked h-BN powders. As can be seen, Si-O bonding was detected on the treated h-BN surface but not on the naked powder, indicating a successful surface treatment of the h-BN powder with GPTMS. This successful surface treatment is also indicated by the TGA analysis, in which the weight losses of the GPTMS-treated h-BN were measured to be 3.132 wt % and 0.825 wt %, greater than that of the naked powders, *i.e.*, 2.588 wt % and 0.575 wt % for particle sizes of 3.6 μm and 10.6 μm, respectively. However, these weight loss differences are noted to be smaller than those reported by other study [16]. Besides, the –OH vibrational absorption (at 3420 cm^−1^, see Figure 1) was marginally detected on the combustion-synthesized h-BN but were clearly detected on a commercially available h-BN reported by Gu *et al.* and Li *et al.* [17,23]. These may be due to differences in the synthesis method used for h-BN: the combustion synthesized h-BN may contain less –OH or active sites on the surface than the commercially available h-BN does, thus adsorbing a lesser amount of GPTMS on its surface.

### 2.2. Optimum Amount of GPTMS for Surface Treatment

**Figure 2:**
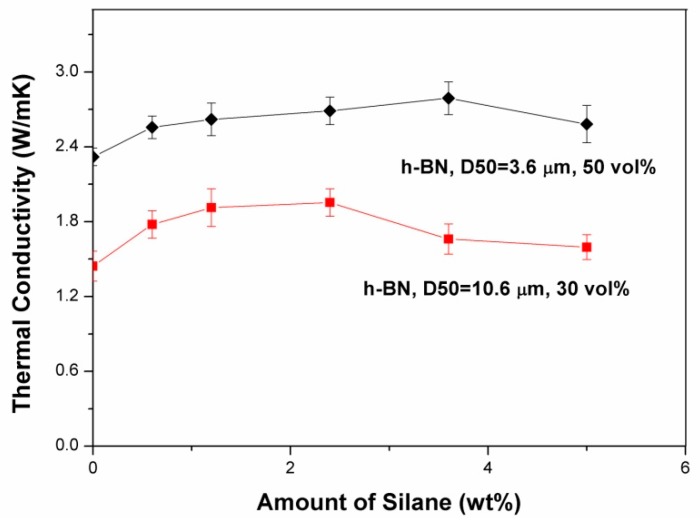
Effect of the amount of GPTMS used in surface treatment of h-BN on the thermal conductivity of the composites.

Figure 2 shows the effect of the amount of GPTMS used in the surface treatment on the thermal conductivity of the composites. The thermal conductivities of the composites are seen to increase with increasing amount of GPTMS to a maximum value (at amounts of 3.6 wt % and 2.4 wt % for particle sizes of 3.6 μm and 10.6 μm, respectively) and then to decrease with further increases in the amount of GPTMS. A similar phenomenon was also reported by Xu *et al.* [16]. Since the GPTMS serves as a coupling agent bridging together the BN particle surface and the matrix molecules (thus reducing the voids at the interface), the thermal conductivity of the composite thus increases with increasing amount of GPTMS (in the region of low amount of GPTMS, *i.e.*, ≤3.6 wt % or ≤2.4 wt % for 3.6 μm or 10.6 μm h-BN particles, respectively). However, the GPTMS at the interface between the BN particles and the matrix also presents as a thermal barrier. When an excess amount of GPTMS was used (*i.e.*, >3.6 wt % or >2.4 wt % for 3.6 μm or 10.6 μm h-BN particles, respectively), this thermal barrier effect dominated over the coupling agent effect (due to a thick GPTMS layer at the interface), thus causing a decrease in the thermal conductivity (these amounts of GPTMS, *i.e.*, 3.6 wt % and 2.4 wt % for 3.6 μm and 10.6 μm h-BN particles, respectively, were thus considered as optimum for surface treatment and were used in all the other experiments presented in this work). It is also noted that the optimum amount of silane for surface treating 3.6 μm h-BN particles is higher than that for 10.6 μm h-BN particles due to the higher specific surface area of the smaller particles.

### 2.3. Effect of Surface Treatment on Thermal Conductivity of Composites

**Figure 3:**
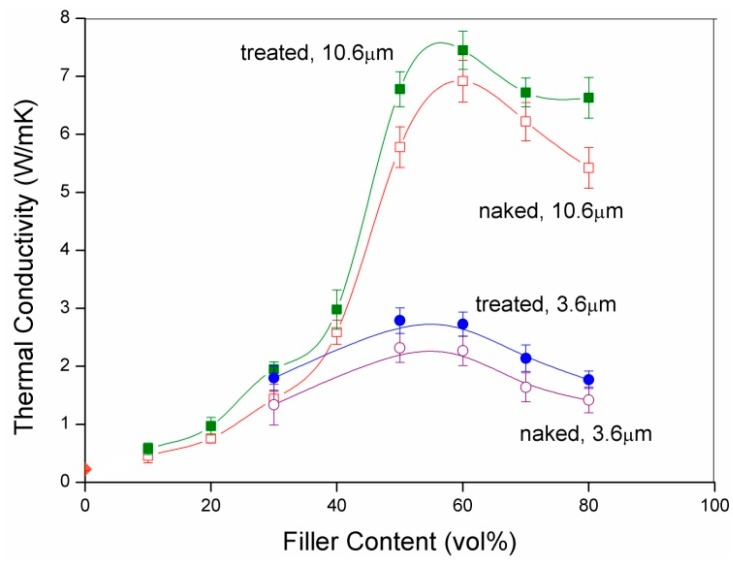
Effects of surface treatment, h-BN particle size and filler content on thermal conductivity of composites.

Figure 3 shows the effect of GPTMS surface treatment on the thermal conductivity of the composites filled with h-BN with an average particle size (d_50_) of 3.6 μm or 10.6 μm. As can be seen, GPTMS surface treatment does increase the thermal conductivity of the composites filled with either small (3.6 μm) or large (10.6 μm) size h-BN particles. As mentioned previously, this increase of thermal conductivity has been explained [16,24] by enhancing bonding between the filler particles and the matrix molecules through bridging by GPTMS, thus reducing the voids and thus the thermal barrier at the filler-matrix interface. As shown by this figure, the fractional increase of thermal conductivity due to GPTMS surface treatment ranges from 15.1% to 20.3%. These fractional increases are comparable to the values reported in reference [16] at low filler contents (e.g., 40 vol %–50 vol %) but are much lower than those reported at high filler contents (*i.e.*, 60 vol % [16]) perhaps due to surface property difference caused by different synthesis methods. Nevertheless, the thermal conductivities of the composites filled with the combustion synthesized h-BN in the present study are found to be comparable to those reported in other studies [16,19,24,25,26,27] where commercially available h-BN was used.

### 2.4. Density and Porosity of Composites

**Figure 4:**
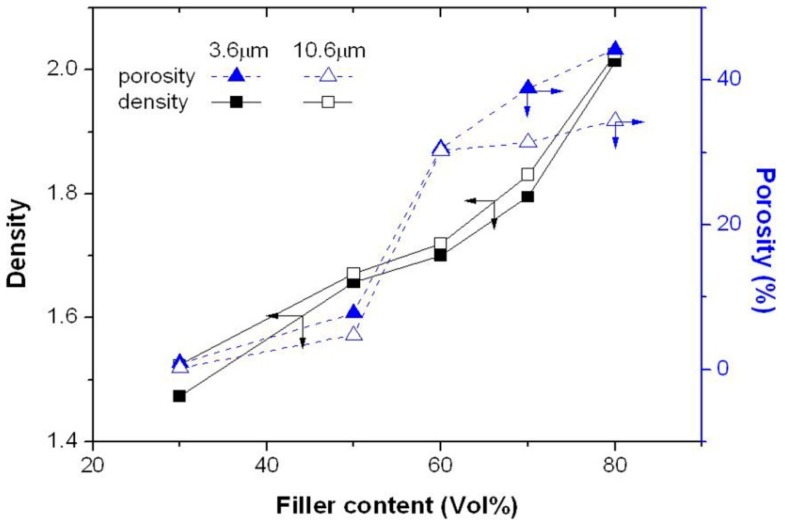
The density and porosity of the epoxy resin composites as a function of filler content.

Figure 4 shows the density and porosity of the epoxy resin composites as a function of filler content. As expected, the density of the composite increases with increasing filler content due to a higher density of the filler compared to that of the epoxy resin. However, the porosity is also seen to increase with increasing filler content, which may be partially due to the fact that the epoxy resin and the filler particles are not completely at close contact at the interface, thus causing a higher porosity at a higher filler content. Besides, the epoxy resin may not be able to fill all the voids formed between the filler particles at high filler contents due to an insufficient amount of epoxy resin or a higher viscosity (resulting in a poor flowability) at high filler contents. This may explain the faster increase of porosity (and the slower increase of density) when the filler content is increased to beyond 50 vol % (see Figure 4).

### 2.5. Effects of h-BN Particle Size and Loading on Thermal Conductivity of Composites

Figure 3 also shows the effects of h-BN particle size and loading on the thermal conductivity of the composites. As can be seen, at each filler content (loading), the thermal conductivity of the composite filled with large particles (d_50_ = 10.6 μm) is higher than that with small particles (d_50_ = 3.6 μm). This is considered to be due to that a composite filled with larger particles has a smaller interface area, and thus a smaller thermal conduction resistance because the interface is believed to contribute the most thermal conduction resistance.

It is also seen in Figure 3 that the thermal conductivity of the composite (filled with either 3.6 μm or 10.6 μm particles with or without surface treatment) increases with increasing filler content to a maximum and then begins to decrease with further increases in filler content. The increase of the thermal conductivity is apparently due to that the thermal conductivity of the filler (*i.e.*, 600 W/mK and 30 W/mK for horizontal and vertical directions of h-BN, respectively) is much higher than that of the matrix (*i.e.*, 0.15–0.25 W/mK for cresol Novolac epoxy resin [7,8]).

The decrease of the thermal conductivity is seen in Figure 3 to begin to occur at a filler loading of ~50 vol %, where the porosity is seen to begin to increase at a faster rate (Figure 4). Since pores (or voids) are a strong thermal barrier, they reduce significantly the thermal conductivity of the composites. Although increasing filler content can increase the thermal conductivity, the porosity effect may dominate at high filler contents. The thermal conductivity of the composites thus begins to decrease with increasing filler content when the filler content is increased to beyond a certain value). The decrease of the thermal conductivity shown in Figure 3 is thus considered to be caused by to the faster increase of the porosity (note that a similar decrease of thermal conductivity of composites has also been observed by many other studies [19,24] and was explained by the fact that at high filler loadings, the matrix is unable to fill in all the voids formed between the filler particles due to insufficient amounts of matrix or/and poor flowability caused by high viscosities at high filler loadings. This enhanced formation of voids was measured in this study to be porosity which increased at a faster rate at high filler loadings).

**Figure 5:**
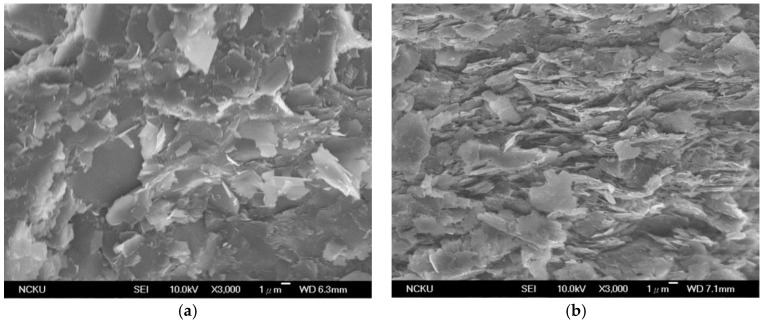
SEM photographs of the fractured surfaces of the composites with filler contents of (**a**) 50 vol % and (**b**) 70 vol % (both composites were filled with 3.6 μm h-BN particles with 3.6 wt % GPTMS treatment).

However, this decrease of thermal conductivity of the composites (with increasing filler content at high filler loadings) was also found in this study to be related to the anisotropy of h-BN in thermal conductivity. Figure 5a,b are two SEM photographs of the fractured surfaces of the composites with filler contents of 50 vol % and 70 vol %, respectively (both composites were filled with 3.6 μm h-BN particles with 3.6 wt % GPTMS treatment). As can be seen, the h-BN particles are more randomly oriented at the low filler content (*i.e.*, 50 vol %) but are more horizontally oriented at the high filler content (*i.e.*, 70 vol %) (this horizontal orientation of the h-BN particles seems to be caused by the pressing which was applied vertically to the specimens during formation of the composites). These horizontally oriented h-BN particles were found to increase with increasing filler content as the filler content was increased to beyond a certain value, *i.e.*, e.g., 50 vol % and 60 vol % when surface-treated 3.6 μm and 10.6 μm h-BN particles were used, respectively. As mentioned previously, the thermal conductivity of h-BN in the vertical direction is much lower than that in the horizontal direction. The thermal conductivity of the composites thus begins to decrease as the filler content is increased to beyond a certain value (*i.e.*, 50 vol % and 60 vol % when surface-treated 3.6 μm and 10.6 μm h-BN particles were used, respectively, in this work; note that the thermal conductivity of the composites was measured in the vertical direction of the specimens in this study).

### 2.6. Thermal Conductivity Model

Many theories have been developed for predicting the thermal conductivity of two-phase systems [28]. Among these, the one developed by Halpin-Tsai [29] and modified by Nielsen and Lewis [30] takes into account the most influencing factors. According to Nielsen and Lewis theory [29], the thermal conductivity is expressed in the general form:

(1) $$K=K_{m}\frac{1+AB\phi }{1-\psi B\phi }$$

where K is the thermal conductivity of the composites and K_m_ that of the matrix. The parameter A depends primarily upon the shape of the dispersed particles and how they are oriented with respect to the direction of the thermal flow and it is related to the generalized Einstein coefficient P_E_ [31] as follows:

A = P_E_ − 1 (2)

where P_E_ accounts for the shape of the particles in suspension and for non-spherical particles, it can be obtained from aspect ratio, p, by following the Guth equation [31]:

P_E_ = {p/[2ln(2p) − 3]} + 2 (3)

The parameter B in Equation (1) takes into account the relative conductivity of the two components:

(4) $$B=\frac{\left(\frac{K_{f}}{K_{m}}\right)-1}{\left(\frac{K_{f}}{K_{m}}\right)+A}$$

where K_f_ and K_m_ are the thermal conductivities of the filler and the matrix, respectively. The parameter ψ in Equation (1) depends on the volume fraction, ϕ, and the maximum packing fraction, ϕ_m_, of the dispersed particles:

(5) $$\psi =1+\frac{1-\phi_{m}}{\phi_{m}^{2}}\;\phi$$

**Figure 6:**
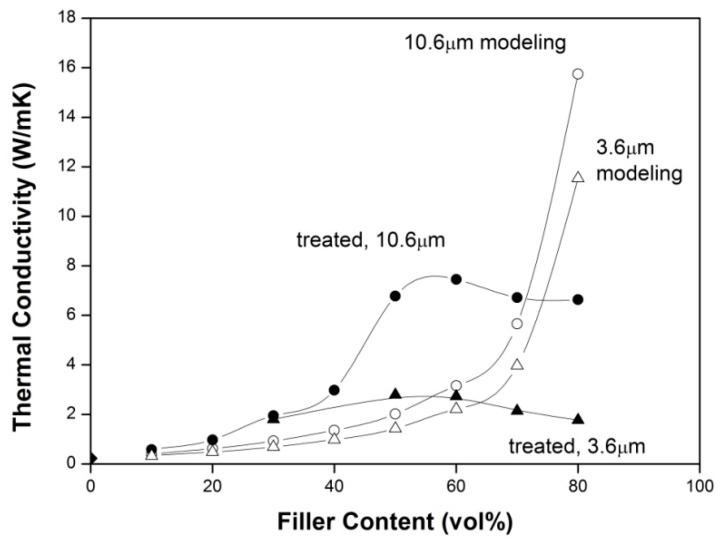
The experimental and predicted thermal conductivity of h-BN/epoxy resin composites with various h-BN contents.

The maximum packing fraction $\phi_{m}$ is defined as the true volume of the particles divided by the volume they occupy when packed to their maximum extent. In the present work, the maximum packing fraction for thin platelets reported by Hill and Supancic [32], *i.e.*, $\phi_{m}$= 0.85, was used. The thickness and the thermal conductivity of the filler were taken as 300 nm and 80 W/mK, respectively. The aspect ratio were taken to be 35 and 12 for 10.6 μm and 3.6 μm h-BN particles, respectively. The resulting values of parameter A and B were 7.41 and 0.98 for 10.6 μm h-BN particles, respectively, and those for 3.6 μm h-BN particles were 4.58 and 0.99, respectively. Predictions are compared to experimental thermal conductivity of h-BN/epoxy resin composites in Figure 6.

As can be seen, at filler contents lower than 60 vol %, the experimental data are higher than the values calculated from the Nielsen and Lewis theory. This may be due to the fact that h-BN particles are highly anisotropic in thermal conductivity but the theory assumes the thermal conductivity of filler particles to be isotropic. At filler loadings higher than 60 vol %, it is seen that the theoretical predictions are increasingly higher than the experimental measurement. This may be due to that the theory does not consider the possible formation of pores or voids at high filler loadings. As mentioned previously, the epoxy resin may not be able to fill all the voids formed between the filler particles at high filler contents due to an insufficient amount of epoxy resin or a higher viscosity (resulting in a poor flowability) at high filler contents. This causes a significant decrease of thermal conductivity thus creating an increasing deviation from the theory with increasing filler content.

## 3. Experimental Section

### 3.1. Materials

The h-BN powder used in this study was prepared in our laboratory by using our newly developed combustion synthesis method [22]. B_2_O_3_, Mg, NH_4_Cl and NaN_3_ were used as the reactants and they were mixed and placed in perforated aluminum containers. The combustion synthesis reaction could be carried out under low N_2_ pressures (<1.0 MPa) with high product yields this process is characterized by low energy consumption, fast reactions, simple processing and low production cost, have also been commercialized for industrial production. The X-ray diffraction pattern of h-BN and the scanning electron micrograph of h-BN powder are presented in Figure S1 and S2 (Supplementary Materials).

To investigate the effect of particle size of the h-BN powder on the thermal conductivity of the epoxy resin composites, the synthesized product was milled to powders with two average particle sizes, *i.e.*, d_50_= 3.6 μm and 10.6 μm. The characteristics of the h-BN powders are listed in Table 1.

The epoxy resin and curing agent used in this study were Novolac epoxy resin (CNE200EL) and phenol Novolac hardener (PF1120HH), respectively, both from Chang Chun Plastics Co., Ltd., (Kaohsinug, Taiwan). 1-Benzyl-2-methylimidazole (90% purity, Sigma Aldrich, St. Louis, MO, USA) was used as catalyst, and γ-glycidoxypropyltrimethoxysilane (97% purity, Acros Organics, Morris, NJ, USA, referred to as GPTMS) was used for surface treatment of the h-BN powders.

### 3.2. Surface Treatment of h-BN Powder

Inorganic particles are usually difficult to disperse in organic polymer matrices because of the poor affinity between them. Surface treatment of inorganic particles is usually carried out, to enhance the affinity and silane molecules are often used as the surface treatment agents (or coupling agent) because they have two functional groups, which of one is reactive with polymers and the other reacts with the particle surface [33]. In this study, surface treatment of h-BN powders was carried out by using GPTMS, a type of silane, as coupling agent.

A proper amount of GPTMS silane (0.6 wt %–5 wt % of h-BN powder used) was added to deionized water (DI, 150 mL) at pH 4.5, into which h-BN (15 g) was added. The solution was heated to 60–70 °C for 20 min and ultrasonication was simultaneously applied to achieve a sufficiently homogeneous dispersion and thus a successful surface treatment. The resultant powder was then obtained by filtration, rinsing with DI water and drying.

### 3.3. Fabrication of h-BN-Filled Epoxy Resin Composites

Listed in Table 2 shows are the components, their functions and their percentage contents used to fabricate the composite specimens. Two processes were employed to obtain homogeneous mixtures of these components: a dry process (involving no use of solvent) and a solvent process (using acetone solvent). The dry process is generally preferred because it involves no use of solvent and is simpler in processing. However, it can only be employed at low filler contents (≤40 vol %) because mixing becomes difficult at high filler contents (>40 vol %). Although the solvent process can achieve homogeneous mixing at both low and high filler contents, highly porous solids were generated (after removing the solvent) at low filler contents (≤40 vol %), which created difficulties in obtaining high density epoxy resin composites. As a consequence, the dry process was employed at low filler contents (≤40 vol %) and the solvent process was employed at high filler contents (>40 vol %).

In the dry process, the epoxy resin (CNE), the hardener (PN) and the catalyst (1-benzyl-2-methylimidazole) were mixed thoroughly by heating them to a liquid state at 120 °C with simultaneous stirring. The h-BN powder was then added to the liquid and the mixture was further heated to 150 °C with simultaneous stirring for at least 10 min to obtain a good dispersion of the h-BN particles in the liquid. After cooling, the solid mixture was crushed to obtain granules under 1 mm for preparation of composite specimens.

In the solvent process, the h-BN powder, CNE, PN and catalyst were added to high purity acetone, and were mixed by mechanical stirring for at least 3 h. The slurry was then dried in a vacuum oven to completely remove the solvent. The solid mixture thus obtained was crushed to obtain granules under 1 mm for fabrication of composite specimens.

To obtain composite specimens, the granules obtained by either the dry or the solvent process were placed in a mold at 175 °C and were uniaxially pressed with a pressure of 50 kg/cm^3^ for 5 min. The compact thus obtained was then placed in a vacuum oven at 180 °C for 6 h for post-curing to enhance cross-linking. The final specimen obtained was cylindrical in shape with a diameter of 12.7–12.8 mm and a length of 1–3 mm.

### 3.4. Characterization of h-BN Powder and h-BN-Filled EMC Epoxy Resin Composites

The particle size distribution and the oxygen content of the h-BN powder were measured by using a particle size analyzer (LA-950V2, Horiba, Kyoto, Japan) and a N/O analyzer (TC 300, Leco, St. Joseph, MI, USA), respectively, and the specific surface area of the h-BN powder was measured by using a BET surface area analyzer (ASAP 2020, Micromeritics, Norcross, GA, USA). The surface elemental composition of particles was analyzed by using X-ray Photoelectron Spectrometry (XPS, Kratos Axis Ultra DLD, Manchester, UK). The FT-IR spectra of the h-BN powders were measured by Fourier transform spectroscopy (Nicolet 6700, Thermo Nicolet Corp., Madison, WI, USA), in a frequency region of 4000–650 cm^−1^ by co-adding 32 scans and at resolution of 4 cm^−1^. The morphology of the h-BN powder and the fractured surface of composite specimens were analyzed by a FESEM (JSM-6700F, JEOL, Tokyo, Japan). The densities of the composite specimens were measured by an Archimedes displacement method. The thermal diffusivity and heat capacity of the composite specimens were measured by a laser flash method (LFA447, Netzch, Selb, Germany). The thermal conductivities of the specimens were obtained by calculation according to the formula: K = Cp × ρ × α, where K is thermal conductivity, Cp is the heat capacity, ρ is the density, and α is the thermal diffusivity [34]. The specific heat capacities were obtained by using Pyroceram 9606 as reference.

## 4. Conclusions

Thermal conductivity of cresol Novolac epoxy (CNE) resin filled with combustion-synthesized h-BN particles was investigated. Surface treatment of the h-BN particles with 3-glycidoxypropyltrimethoxysilane (GPTMS) was found to increase the thermal conductivity of the composites by 7.7%–35.4%, which is less than a value reported in another study at a high filler content. This was explained by the fact that the combustion synthesized h-BN contains less –OH or active sites on the surface, thus adsorbing less amount of GPTMS. However, the thermal conductivity of composites filled with the combustion synthesized h-BN were found to comparable to that with commercially available h-BN reported in other studies. The thermal conductivity of the composites was found to be higher when larger h-BN particles were used. The thermal conductivity was also found to increase with increasing filler content to a maximum and then begin to decrease with further increases in this content. In addition to the effect of higher porosity at higher filler contents, more horizontally oriented h-BN particles formed at higher filler loadings (perhaps due to pressing during formation of the composites) were suggested to be a factor causing this decrease of the thermal conductivity. The measured thermal conductivities were compared to theoretical predictions based on the Nielsen and Lewis theory. The theoretical predictions were found to be lower than the experimental values at low filler contents (<60 vol %) and became increasing higher than the experimental values at high filler contents (>60 vol %).

## Figures and Tables

**Table 1:** The Characteristics of the h-BN Powders.

Material	Mean Size (μm)	D_10_ (μm)	D_50_ (μm)	D_90_ (μm)	Oxygen Content (wt %)	Specific Surface Area (m^2^/g)	Impurity
h-BN	3.6	2.04	3.64	6.39	2.63	35.83	<3.52
h-BN	10.6	5.93	10.67	20.96	1.46	9.33	<2.34

**Table 2:** Components, their functions and their percentage contents used to fabricate the composite specimens.

Component (Function)	Compound or Commercial Name of Component	Physical State at Room Tempersture	Percentage Contents (wt %)
Filler	h-BN powder	solid	16–87.5
Matrix	Cresol Novolac epoxy resin (CNE)	solid	8–55
Hardener	Phenol Novolac (PN)	solid	4–28
Catalyst	1-Benzyl-2-methylimidazole	liquid	0.02
Coupling agent	3-Aminopropyltriethoxysilane	liquid	0.5

## Supplementary Materials

Supplementary materials can be accessed at: http://www.mdpi.com/1420-3049/21/5/670/s1.
