# Supplementary material for: Thermal Conductivity of Epoxy Resin Composites Filled with Combustion Synthesized h-BN Particles
Source: Molecules. 2016 May 20;21(5):670. doi: 10.3390/molecules21050670 (PMC6273151; doi:10.3390/molecules21050670)
Supplement: Supplementary file 1 [file molecules-21-00670-s001.pdf]

Supplementary Materials: Thermal Conductivity of Epoxy Resin Composites Filled with Combustion Synthesized h-BN Particles

Shyan-Lung Chung and Jeng-Shung Lin

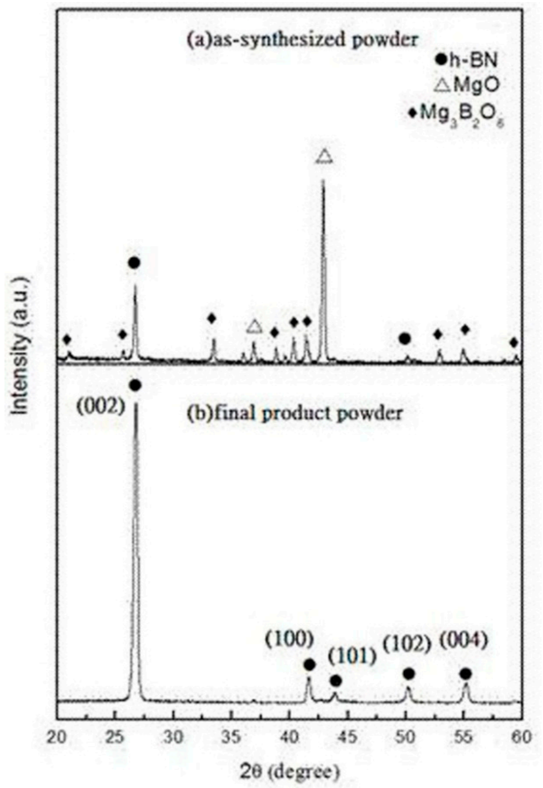

Figure S1. The X-ray diffraction pattern of h-BN (**a**) as-synthesized powder (**b**) final product powder.

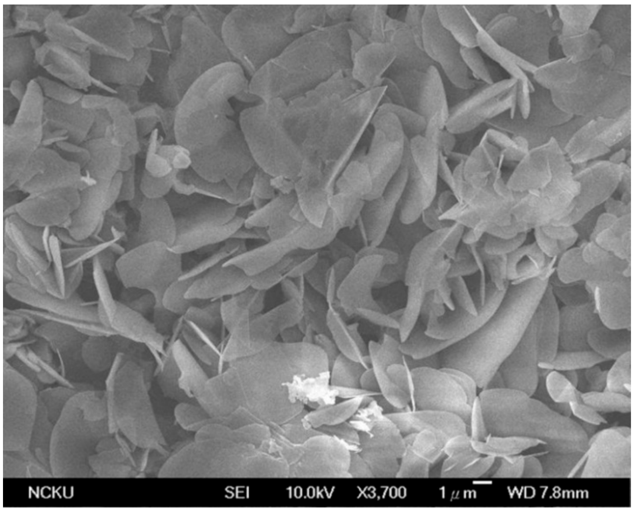

Figure S2. The scanning electron micrograph of h-BN powder with an average particle size of 10.6 μm.

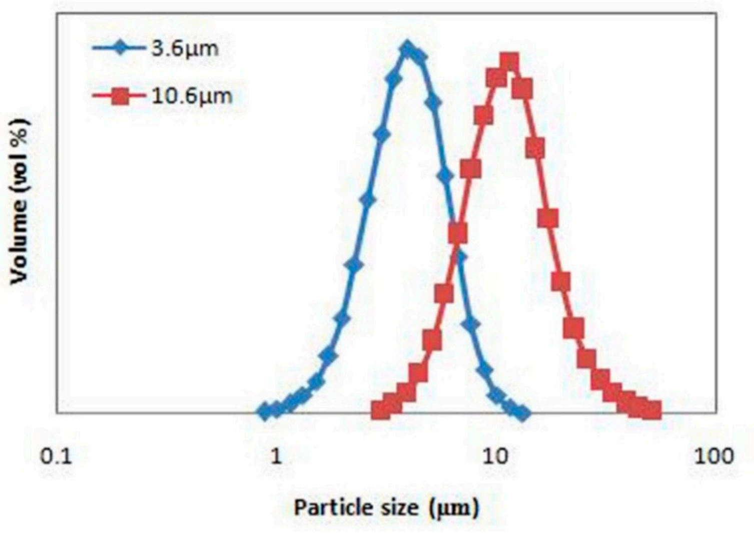

Figure S3. Particle size distribution of h-BN powder.

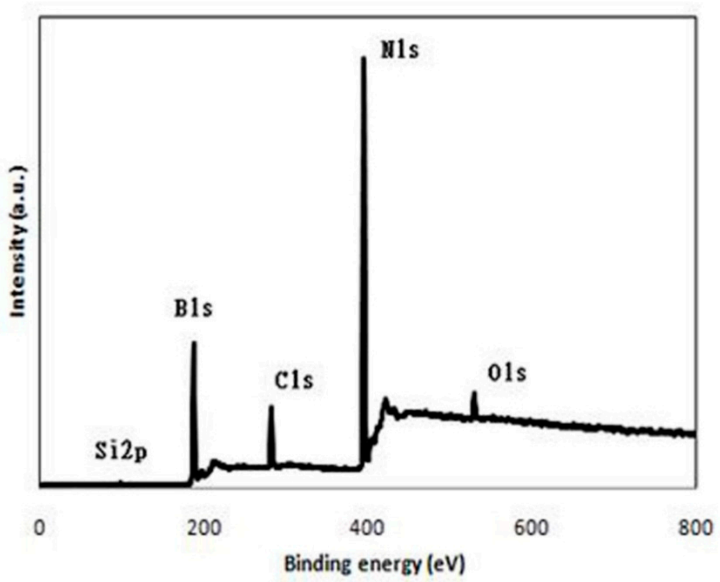

Figure S4. X-ray photoelectron spectroscopy of 2.4 wt % GPTMS-treated h-BN powders with an average particle size of 10.6 µm.

Table S1. Charge corrected binding energies (eV) and possible assignment for silane GPTMS treated h-BN.

Energy level	Binding Energy (eV)	Assignment
C_{1s}	285.0	$(CH_2)_n$
	287.2	C-O
Si_{2p}	102.1	Organic silicone
B_{1s}	189.8	BN
O_{1s}	531.8	Si-O
N_{1s}	397.8	BN

Table S2. Fractional loss in weight of h-BN particles upon heating to 600 °C.

Particle Size	3.6 μm	10.6 μm
Naked powder	2.83%	0.68%
Silane treated	3.25%	0.83%

Table S3. The thermal conductivity of EMCs with various filler contents and particle sizes (with and without surface treatment).

Particle Size, d50 (μm)	Filler Content (vol %)	10	20	30	40	50	60	70	80
	native	0.46	0.76	1.44	2.59	5.78	6.92	6.22	5.42
10.6	treated	0.58	0.97	1.95	2.98	6.78	7.45	6.72	6.63
	ΔK %	26.1	27.6	35.4	15.1	17.3	7.7	8.0	22.3
	native	-	-	1.34	-	2.32	2.27	1.64	1.42
3.6	treated	-	-	1.80	-	2.79	2.73	2.14	1.77
	ΔK %	-	-	34.3	-	20.3	20.3	30.5	24.6

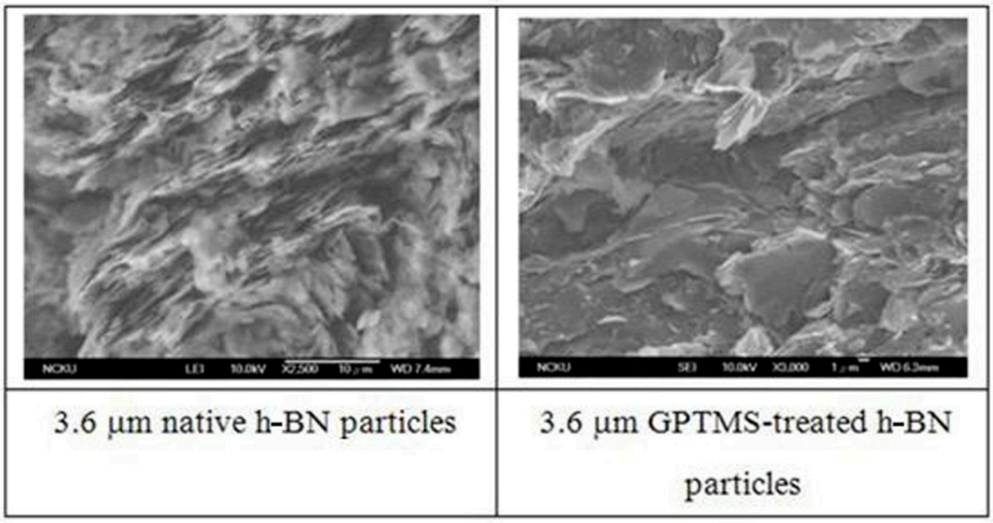

Figure S5. SEM micrographs of cross-section of 3.6 wt % GPTMS-treated h-BN epoxy-matrix composites with an average particle size of 3.6 μm (without polishing or etching).
